# Five-Year Outcomes of Trabeculectomy and Phacotrabeculectomy

**DOI:** 10.7759/cureus.12950

**Published:** 2021-01-27

**Authors:** Danny Lam, David Z Wechsler

**Affiliations:** 1 Ophthalmology, University of Sydney, Sydney, AUS; 2 Ophthalmology, Macquarie University, Sydney, AUS

**Keywords:** trabeculectomy, phacotrabeculectomy, intraocular pressure

## Abstract

Purpose

The purpose of this study is to examine five-year outcomes of trabeculectomy and compare the stand-alone procedure when combined with phacoemulsification.

Patients and methods

This study included 123 eyes of 109 patients, with 79 patients in the trabeculectomy group and 44 patients in the phacotrabeculectomy group. Non-randomized comparative cohort study with data collected retrospectively from an existing database compiled by a single surgeon operating in Sydney, Australia from 2007 to 2019. The primary outcome measure was intraocular pressure. Secondary outcome measures were a number of glaucoma medications, treatment success rates, best-corrected visual acuity, bleb morphology, post-operative complications, and re-operation rate.

Results

The mean intraocular pressure was 10.6 ± 2.7 mm Hg in the trabeculectomy group (pre-operative mean intraocular pressure of 28.0 ± 9.8) and 12.0 ± 3.0 mm Hg in the phacotrabeculectomy group (pre-operative mean intraocular pressure of 23.4 ± 7.9) after five years (P = 0.052). The number of glaucoma medications required was 0.3 ± 0.7 in the trabeculectomy group (pre-operative mean of 3.7 ± 1.1) and 1.3 ± 1.2 in the phacotrabeculectomy group (pre-operative mean of 3.1 ± 1.0, P < 0.001).

Conclusions

Intraocular pressure reduction post-operatively over five years was similar between trabeculectomy and phacotrabeculectomy as determined by mean intraocular pressure, and intraocular pressure reduction from baseline. However, fewer supplemental glaucoma medications were required following trabeculectomy as compared to the combined procedure.

## Introduction

Trabeculectomy is the established gold standard in glaucoma filtering surgery [1]. The optimal surgical technique for performing trabeculectomy has undergone many refinements with no national guidelines or preferred practice for the procedure [2]. Trabeculectomy can be associated with significant intra-operative and post-operative complications. A major limitation of trabeculectomy resulting in surgical failure is scarring. However, success rates have been greatly improved with the use of the anti-fibrotics mitomycin C (MMC) and 5-fluorouracil (5-FU) intra-operatively and post-operatively [3]. A longer-term sequela of trabeculectomy is the acceleration of cataract formation and progression [4]. While the risk of developing cataract is present with increasing age regardless of surgical intervention, the Advanced Glaucoma Intervention Study quantified the increased risk of cataract formation after a trabeculectomy compared to without trabeculectomy to be 78% [4]. In patients with coexistent glaucoma and cataract, there is the option of performing cataract surgery (phacoemulsification) combined with trabeculectomy [5].

Phacotrabeculectomy has been advocated as an effective treatment for coexisting cataract and glaucoma [6]. However, the convenience of the combined procedure may be at the expense of decreased success rates for intraocular pressure (IOP) control. The amplified inflammatory response of phacotrabeculectomy may increase the chance of filtration bleb scarring, resulting in poorer long-term filtration [5]. There are also the associated risks of cataract surgery, and if complicated by vitreous loss this could further compromise the filtration procedure [5].

There is a number of studies comparing trabeculectomy with other glaucoma procedures, such as the Tube Versus Trabeculectomy (TVT) Study and Primary Tube Versus Trabeculectomy (PTVT) Study [7,8]. The literature comparing the relative efficacy of trabeculectomy and phacotrabeculectomy is scarce with published studies having short follow up periods (generally 1-2 years) or small sample sizes (mostly less than 100 total patients) [5,6,9-13]. This study aims to compare the outcomes over five years of trabeculectomy and phacotrabeculectomy, examining IOP outcomes and success rates, as well as the incidence of post-operative complications, and the need for post-operative interventions.

## Materials and methods

The Research and Ethics offices at Concord General Repatriation Hospital and Liverpool Hospital approved the research protocol prior to data collection. The study was conducted in accordance with the National Statement on Ethical Conduct in Human Research, and consistent with the principles that have their origin in the Declaration of Helsinki.

Data source

The data collected in this study were extracted from an existing database of 269 patients of a single surgeon operating at two tertiary referral teaching hospitals (Concord General Repatriation Hospital and Liverpool Hospital, Sydney, NSW) and two private hospitals (Sydney Private Hospital and Macquarie University Hospital, Sydney NSW) from 2007 until 2019. The data had been previously collected in a contemporaneous fashion as part of an ongoing audit of patients undergoing glaucoma surgery (trabeculectomy or phacotrabeculectomy).

Eligibility criteria

Patients with primary open-angle glaucoma, angle-closure glaucoma, normal-tension glaucoma, and pseudoexfoliation glaucoma who had undergone trabeculectomy or phacotrabeculectomy with at least five years of follow-up data were included in the study. Exclusion criteria were patients who had their procedure less than five years ago, patients who have had previous ophthalmic procedures, and patients with secondary glaucoma associated with increased risk of trabeculectomy failure including uveitic, pigmentary, traumatic, and neovascular glaucoma. Because these eyes are recognized to be at increased risk of surgical failure, they were unlikely to be offered phacotrabeculectomy in any case, and thus would be unhelpful in comparing the two procedures [14]. Since eyes with exfoliation syndrome tend to do well with trabeculectomy, and by extension phacotrabeculectomy, this was the one secondary glaucoma that was eligible for inclusion [15]. Both eyes of eligible patients were included in the study if they otherwise met the inclusion criteria.

Patient visits

Baseline demographic information collected includes age, gender, race, past medical and surgical history. Baseline ocular information collected included glaucoma diagnosis, operated eye, best-corrected visual acuity (BCVA), IOP, presence of visual field defect and mean deviation (MD) on visual field testing, number of glaucoma medications, lens status, and previous ocular surgery.

Post-operative data collected at one year, two years, and five-year time points were BCVA, IOP, a number of glaucoma medications, complications, need for re-operation, and bleb morphology. The one and two-year time points were selected to show shorter-term progress and the five-year time point was selected to show longer-term progress.

Outcome measures

The primary outcome measure was IOP. In addition, a target IOP was determined clinically based on the patient’s stage of glaucoma pre-operatively and was not changed depending on the procedure. When post-operative IOP data were collected at the various time points during follow-up, a note was made whether target IOP was achieved in addition to the numerical IOP value.

Secondary outcome measures included a number of glaucoma medications, treatment success rates, best-corrected visual acuity, bleb morphology outcome, post-operative complications, and re-operations.

The criterion for decreased visual acuity was a decrease in two or more lines of Snellen acuity from baseline at the post-operative time points. Treatment failure was defined as not achieving the desired target IOP, repeat operation for glaucoma (excluding bleb needling), or the development of serious complications. Absolute success was defined as achieving the target IOP without glaucoma medications or vision-threatening complications. Qualified success was defined as achieving the target IOP with the additional use of glaucoma medications. Bleb morphology was documented via the Indiana Bleb Appearance Grading Scale [16]. Further re-operations documented include needling, repeat glaucoma surgery, surgery for post-operative complications, and cataract surgery.

Statistical analysis

Univariate comparisons between treatment groups were performed with the two-sided Student t-test for continuous variables. A P-value of 0.05 or less was considered statistically significant in the analysis. Treatment comparisons of the cumulative proportion of treatment success were assessed with the stratified Kaplan-Meier survival analysis log-rank (Mantel-Cox) test using the GraphPad Prism^TM^ software (GraphPad Software, CA).

Surgical methods

All surgeries were performed by or under the supervision of a single surgeon. The selection of patients to have a combined procedure was determined by the surgeon, taking into consideration the degree of visual impairment from the cataract, co-existent narrow-angle component attributable to the cataract, and pre-operative assessment of the risk of trabeculectomy failure.

Surgery was generally performed under local anesthesia with peribulbar or Sub-Tenon’s block, and in some cases under general anesthesia depending on patients’ preferences and ability to tolerate the procedure. A fornix-based conjunctival flap was used for all cases, with a rectangular 5-6 x 3-4 mm half-thickness scleral flap. An anti-fibrotic regimen was selected based on the anticipated risk of postoperative scarring. Most patients received MMC applied on five sponges into the subconjunctival space after the scleral flap was fashioned but prior to entry into the anterior chamber. Doses of 0.2-0.4 mg/ml were used for 2-3 mins. Some cases with minimal risk factors for post-operative scarring received 5-FU on sponges (50 mg/ml) rather than MMC. All cases of combined phacotrabeculectomy received MMC. An anterior chamber maintainer was used for most cases of standalone trabeculectomy. A single punch osteum with Khaw punch^R^ (750 µm) was used to create the osteum under the scleral flap and surgical iridectomy was performed with Vannas scissors. A combination of preplaced 10-0 nylon releasable and adjustable sutures was used to close the scleral flap. The conjunctiva was closed with 10-0 nylon sutures at the limbus and 8-0 vicryl sutures on either side if relaxing incisions in the peritomy were required during surgery.

In cases of combined phacotrabeculectomy, a 2-site approach was used with a separate temporal clear corneal incision for the phacoemulsification with a posterior chamber lens implant. A standard one-piece acrylic hydrophilic monofocal lens was implanted in the capsular bag for all cases. The corneal wound was sutured with 10-0 nylon and ensured to be watertight, and the pupil was constricted with acetylcholine chloride intraocular solution (Miochol^TM^) prior to performing the trabeculectomy. 

All patients received a drop of atropine 1% at the end of the case. Post-operatively patients were treated with chloramphenicol drops four times a day for one week, and intensive topical dexamethasone 0.1% eye drops four times a day for over six to eight weeks. Drop frequency and duration were titrated to the degree of conjunctival vascularity and inflammation observed at post-operative visits.

## Results

Recruitment and retention

Out of 269 total patients in the database, 160 patients were excluded due to having their procedure within the last five years (with less than five years of follow-up data available) or having a diagnosis of secondary glaucoma. A total of 123 eyes of 109 patients was included in the study, with 79 eyes in the trabeculectomy group and 44 eyes in the phacotrabeculectomy group.

Loss of data at the one year, two years, or five years post-operative time point was either due to loss to follow up (30 total patients), death (eight total patients), failure of the procedure requiring repeat glaucoma surgery (14 total patients) or switch to medical therapy (seven total patients).

Patients who were lost to follow-up were excluded from the analysis. Those who required repeat operation for glaucoma or where data were no longer collected as they no longer had functioning trabeculectomies were included as eligible patients to determine overall success rates.

Pre-operative characteristics of the study group

The pre-operative and ocular characteristics of the study groups are provided in Table 1. There was a statistically significant difference in age between the trabeculectomy and phacotrabeculectomy groups (P < 0.001). The mean pre-operative IOP of the overall study group was 26.38 ± 9.11 mm Hg and the mean number of glaucoma medications was 3.46 ± 1.09. There was a statistically significant difference in mean pre-operative IOP and number of glaucoma medications between the trabeculectomy and phacotrabeculectomy groups. Eighty-one eyes (66%) were phakic prior to surgery. Forty-two eyes (53%) in the trabeculectomy group were pseudophakic.

**Table 1:** Pre-Operative Characteristics of Trabeculectomy Versus Phacotrabeculectomy Study Groups SD = standard deviation; POAG = primary open-angle glaucoma; PXF = pseudoexfoliation glaucoma; ACG = angle closure glaucoma; NTG = normal tension glaucoma; OHT = ocular hypertension; BCVA = best-corrected visual acuity; IOP = intraocular pressure; VF = visual field; MD = mean deviation

Characteristic	Trabeculectomy (n = 79)	Phacotrabeculectomy (n = 44)	P-value
Age (years)			
Mean ± SD	67.2 ± 12.8	76.4 ± 9.5	< 0.001
Median (range)	68 (35-93)	78 (39-89)	
Gender (%)			
Male	49 (62)	26 (59)	
Female	30 (38)	18 (41)	
Race (%)			
Arabic	7 (9)	3 (7)	
Asian	12 (15)	7 (16)	
Caucasian	50 (63)	32 (73)	
Indian	8 (10)	1 (2)	
Other	2 (3)	1 (2)	
Diagnosis (%)			
POAG	52 (66)	26 (59)	
PXF	19 (24)	9 (20)	
ACG	3 (4)	5 (11)	
NTG	5 (6)	3 (7)	
OHT	0	1 (2)	
Study eye (%)			
Right	44 (56)	21 (48)	
Left	35 (44)	23 (52)	
Pre-operative BCVA (%)			
6/6 or better	20 (25)	5 (11)	
6/9 - 6/18	46 (58)	31 (70)	
Worse than 6/18	13 (16)	8 (18)	
Pre-operative IOP			
Mean ± SD	28.0 ± 9.8	23.4 ± 7.9	0.009
Pre-determined target IOP mean ± SD	12.5 ± 2.3	12.3 ± 2.0	
VF defect (%)			
Y	70 (89)	31 (70)	
N	9 (11)	13 (30)	
MD			
Mean ± SD	-18.5 ± 11.7	-15.0 ± 8.0	0.142
Number of glaucoma medications			
Mean ± SD	3.7 ± 1.1	3.1 ± 1.0	0.009
Phakic/pseudophakic			
Phakic	37 (47)	44 (100)	
Pseudophakic	42 (53)	0	
Operating surgeon			
Consultant	69 (87)	27 (61)	
Trainee	10 (13)	17 (39)	

Intraocular pressure

Both surgical procedures resulted in a significant and sustained reduction in IOP (Table 2). Mean post-operative IOP was lower in the trabeculectomy group compared with the phacotrabeculectomy group, however, the difference was not statistically significant at any follow-up time point.

**Table 2:** Intraocular Pressure and Medical Therapy at Baseline and Follow-Up Data are presented as mean ± standard deviation or as number (percentage). IOP = intraocular pressure *Cases that required repeat operation for glaucoma were excluded.

	Trabeculectomy	Phacotrabeculectomy	P-value
Pre-operative			
Mean IOP (mmHg)	28.0 ± 9.8	23.4 ± 7.9	0.009
Glaucoma medications	3.7 ± 1.1	3.1 ± 1.0	0.009
Number of patients	79	44	
1 year			
Mean IOP (mmHg)	12.2 ± 4.5	12.3 ± 4.0	0.888
IOP at target (%)	41 (57)	21 (57)	
Glaucoma medications	0.4 ± 0.9	0.5 ± 1.0	0.849
Number of eligible patients*	72	37	
2 years			
Mean IOP (mmHg)	11.8 ± 3.2	11.9 ± 3.6	0.633
IOP at target (%)	36 (51)	21 (60)	
Glaucoma medications	0.5 ± 1.0	0.6 ± 1.2	0.559
Number of eligible patients*	70	35	
5 years			
Mean IOP (mmHg)	10.6 ± 2.7	12.0 ± 3.0	0.052
IOP at target (%)	31 (53)	14 (52)	
Glaucoma medications	0.3 ± 0.7	1.3 ± 1.2	< 0.001
Number of eligible patients*	58	27	

Treatment outcomes

Absolute and qualified success rates are presented in Table 3 with absolute success being defined as requiring no glaucoma medications and qualified success being defined as requiring glaucoma medications. The proportion of absolute success and qualified success remained relatively constant at all follow-up points for the trabeculectomy group. The rate of absolute success was similar between the two groups at one year and two years. However, this was significantly higher in the trabeculectomy group compared to the phacotrabeculectomy group at five years (P < 0.001, logistic regression analysis adjusted for stratum).

**Table 3:** Rate of Absolute and Qualified Success for Target Intraocular Pressure Criterion Data are presented as number (percentage). The definitions of absolute success and qualified success are defined above in “Outcome Measures”. Patients with treatment failure are still included in the total for subsequent time slot analyses.

	Trabeculectomy	Phacotrabeculectomy	P-Value
1 year	n = 72	n = 37	
Absolute success	38 (53)	19 (51)	0.779
Qualified success	3 (4)	2 (5)	
Total success	41 (57)	21 (57)	
2 years	n = 70	n = 35	
Absolute success	33 (47)	16 (46)	0.101
Qualified success	3 (4)	5 (14)	
Total success	36 (51)	21 (60)	
5 years	n = 58	n = 27	
Absolute success	29 (50)	5 (19)	<0.001
Qualified success	2 (3)	9 (33)	
Total success	31 (53)	14 (52)	

**Figure 1:**
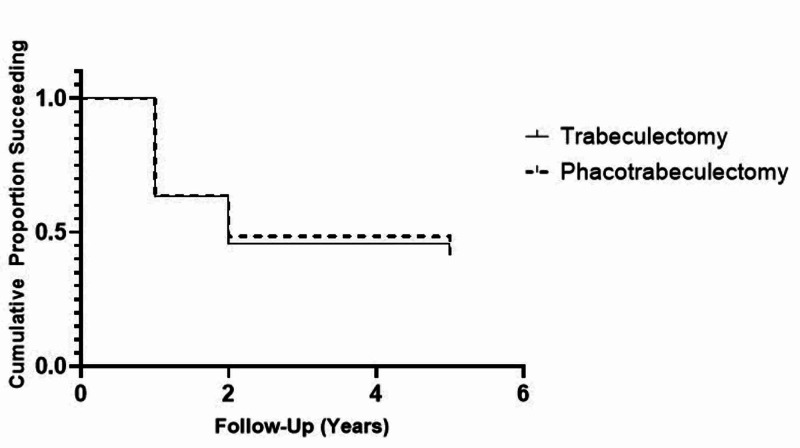
Cumulative Probability of Achieving Target Intraocular Pressure Kaplan-Meier plot showing cumulative probability of successfully achieving the pre-determined target intraocular pressure (IOP).

Kaplan-Meier survival analysis showing absolute success rates for achieving target IOP with trabeculectomy and phacotrabeculectomy at each of the follow-up points are presented in Figure 1. The cumulative probability of success at the endpoint of the study was 42.4% in the trabeculectomy group and 41.6% in the phacotrabeculectomy group (P = 0.998).

The overall failure rate was very similar between the trabeculectomy group and the phacotrabeculectomy group at all follow-up time points. Table 4 lists the reasons for classification as treatment failure. Not achieving target IOP was the most common reason for treatment failure throughout the first five years of follow-up in both treatment groups. There were no treatment failures due to the development of serious complications.

**Table 4:** Reasons for Treatment Failure Data are presented as number (percentage). Patients with treatment failure are still included in the total for subsequent time slot analyses. IOP = intraocular pressure

	Trabeculectomy	Phacotrabeculectomy
1 year	n = 72	n = 37
Target IOP not achieved	26 (36)	13 (35)
Repeat operation for glaucoma	3 (4)	2 (5)
Development of serious complications	0	0
2 years	n = 70	n = 35
Target IOP not achieved	25 (36)	11 (31)
Repeat operation for glaucoma	6 (9)	2 (6)
Development of serious complications	0	0
5 years	n = 58	n = 27
Target IOP not achieved	12 (21)	6 (22)
Repeat operation for glaucoma	9 (16)	5 (19)
Development of serious complications	0	0

Medical therapy

There was a reduction in the use of glaucoma medications in both treatment groups at all post-operative follow-up time points (Table 2). There were fewer medications required in the trabeculectomy group compared with the phacotrabeculectomy group at five years (P < 0.001).

Visual acuity

Pre-operative BCVA for both groups is reported in Table 1. The rate of decrease in VA by two or more lines was similar in the trabeculectomy and phacotrabeculectomy groups at all post-operative follow-up reviews (Table 5).

**Table 5:** Decrease in Visual Acuity by Two or More Lines Data are presented as number (percentage, total number).

Post-Operative Year	Trabeculectomy	Phacotrabeculectomy
1	6 (9%, n = 67)	3 (9%, n = 34)
2	10 (16%, n = 61)	2 (6%, n = 32)
5	5 (12%, n = 43)	1 (5%, = 20)

Bleb morphology

Bleb morphology results, as documented via the Indiana Bleb Appearance Grading Scale, are shown in Table 6. There were consistently more eyes with a bleb height and extent greater than two in the trabeculectomy group compared to the phacotrabeculectomy group at all follow-up time points. At two years and five years, the eyes in the trabeculectomy group had a significantly higher and more extensive bleb than the eyes in the phacotrabeculectomy group. There was a low proportion of avascular and avascular cystic blebs in both surgical groups at all follow-up time points.

**Table 6:** Bleb Morphology Data are presented as number (percentage). H = bleb height; E = horizontal extent; V = vascularity

	Trabeculectomy	Phacotrabeculectomy	P-value
1 year	n = 72	n = 37	
H ≥ 2	51 (71)	21 (57)	0.253
E ≥ 2	59 (82)	29 (78)	0.531
V0 (Avascular white)	3 (4)	1 (3)	
V1 (Avascular cystic)	0 (0)	1 (3)	
2 years	n = 70	n = 35	
H ≥ 2	54 (77)	19 (54)	0.012
E ≥ 2	59 (84)	27 (77)	0.024
V0 (Avascular white)	1 (1)	2 (6)	
V1 (Avascular cystic)	3 (4)	2 (6)	
5 years	n = 58	n = 27	
H ≥ 2	32 (55)	5 (19)	< 0.001
E ≥ 2	37 (64)	13 (48)	0.013
V0 (Avascular white)	3 (5)	0 (0)	
V1 (Avascular cystic)	0 (0)	0 (0)	

Post-operative interventions

Post-operative interventions were performed in 63 patients (80%) in the trabeculectomy group and 35 patients (80%) in the phacotrabeculectomy group and are listed in Table 7.

**Table 7:** Post-Operative Interventions Data are presented as number (percentage). 5-FU = fluorouracil

	Trabeculectomy (n = 79)	Phacotrabeculectomy (n = 44)
Laser suture lysis	18 (23)	10 (23)
5-FU injection	13 (16)	8 (18)
Removal of releasable suture	4 (5)	0 (0)
Needling on slit lamp	4 (5)	0 (0)
Needling in theatres	32 (41)	16 (36)
Anterior chamber reformation	3 (4)	6 (14)
Suture adjust	18 (23)	10 (23)
Bleb massage	28 (35)	21 (48)
Re-suture conjunctiva	2 (3)	2 (5)
Re-suture flap	1 (1)	5 (11)
Total number of patients with post-operative interventions	63 (80)	35 (80)
Cataract surgery	16 (20)	0 (0)

Surgical complications

Table 8 lists the surgical complications throughout five years of follow-up. The total percentage of intra-operative complications was the same between the trabeculectomy and phacotrabeculectomy groups. These complications were of a minor nature. Conjunctival dehiscence/buttonhole was the most common intraoperative complication in both surgical groups occurring in five patients (6%) in the trabeculectomy group and two patients (5%) in the phacotrabeculectomy group. There were otherwise no intraoperative complications with higher frequency in either surgical group. There were no serious intra-operative complications in either group.

**Table 8:** Surgical Complications Data are presented as number (percentage). Data is censored after a repeat operation for glaucoma. Some patients experienced more than one complication. Early post-operative complications had an onset before one month. Late post-operative complications had an onset after one month. Some patients experienced both early and late post-operative complications.

	Trabeculectomy (n = 79)	Phacotrabeculectomy (n = 44)
Intraoperative Complications		
Conjunctival dehiscence/buttonhole	5 (6)	2 (5)
Iris prolapse	1 (1)	1 (2)
AC collapse	1 (1)	1 (2)
Total	7 (9)	4 (9)
Early Post-Operative Complications		
Wound leak	6 (8)	2 (5)
Shallow AC	4 (5)	3 (7)
Epitheliopathy	2 (3)	0 (0)
Cystic macular edema	0 (0)	1 (2)
Filamentary keratitis	4 (5)	0 (0)
Hyphema	1 (1)	0 (0)
Choroidal effusion	3 (4)	5 (11)
Clot in osteum	4 (5)	0 (0)
Extending bleb	2 (3)	0 (0)
Corneal edema	1 (1)	1 (2)
Total	21 (27)	11 (25)
Late Post-Operative Complications		
Dysaesthesia	1 (1)	0 (0)
Inclusion cysts	2 (3)	0 (0)
Corneal edema	3 (3)	0 (0)
Choroidal effusion	1 (1)	1 (2)
Epitheliopathy	1 (1)	0 (0)
Shallow AC	1 (1)	0 (0)
Encapsulated bleb (full/partial)	11 (14)	3 (7)
Total	18 (23)	3 (7)

Early post-operative complications were defined as those developing within the first month post-operatively and the total incidence was again the same between the trabeculectomy and phacotrabeculectomy groups. A total of 27 early post-operative complications was reported in 21 patients (27%) in the trabeculectomy group and a total of 12 complications was reported in 11 patients (25%) in the phacotrabeculectomy group. Wound leak (8%) was the most common early post-operative complication in the trabeculectomy group and choroidal effusion (11%) was the most common early post-operative complication in the phacotrabeculectomy group.

Overall late post-operative complications occurring more than one month after surgery was higher in the trabeculectomy group compared to the phacotrabeculectomy group. A total of 18 patients (23%) had late post-operative complications in the trabeculectomy group and only three patients (7%) had complications in the phacotrabeculectomy group.

Other post-operative surgical events occurring over the five years of follow up include one patient requiring surgical iridectomy for partial obstruction of the osteum with iris incarceration, two patients with corneal decompensation requiring Descemet’s stripping automated endothelial keratoplasty (DSAEK), and one patient requiring anti-vascular endothelial growth factor (anti-VEGF) therapy for age-related macular degeneration (AMD).

Repeat operation for glaucoma

The rate of repeat operation for glaucoma after the failure of the initial surgical procedure was similar in both groups and shown in Table 4. These patients went on to have tube implant surgery (Baerveldt^TM^ device {Johnson & Johnson Vision, CA}).

Cataract progression

Out of the 37 phakic patients in the trabeculectomy group, 16 (43%) underwent phacoemulsification cataract extraction within five years. There were no significant complications associated with phacoemulsification in either the trabeculectomy group or the phacotrabeculectomy group. Only two out of the 16 patients (13%) in the trabeculectomy group who underwent phacoemulsification experienced a decrease in VA of two or more lines on the Snellen chart prior to the procedure that was attributable to cataract.

## Discussion

This study found that trabeculectomy and phacotrabeculectomy were both similarly effective at sustainably lowering IOP over five years. Trabeculectomy lowered the mean IOP by 56.4% at one year, and phacotrabeculectomy by 47.4%. This reduction was sustained at the five-year follow-up point, with trabeculectomy having an average reduction in IOP of 62.1% and phacotrabeculectomy an average reduction in IOP of 48.8%. Additionally, the cumulative probability of successfully achieving the target IOP without medications was 42.4% in the trabeculectomy group and 41.6% in the phacotrabeculectomy at five years.

There was no significant difference in the overall success rate of both surgical procedures at any follow-up time. However, the rate of absolute success in the trabeculectomy group was significantly higher compared with the phacotrabeculectomy group at five years, with a higher number of glaucoma medications being required in the phacotrabeculectomy group to achieve target pressure.

The overall rate of treatment failure was similar in both surgical groups and the reasons for failure were also similar. Patients with trabeculectomy failure requiring further glaucoma surgery typically underwent insertion of a glaucoma drainage device. The decision to re-operate was at the discretion of the surgeon, which raises the possibility of bias in the decision for repeat glaucoma surgery, however, the re-operation rates were similar between the two groups.

Post-operative interventions were performed with a similar rate in both trabeculectomy and phacotrabeculectomy groups. The most common intervention in both surgical groups was the needling of the bleb in the operating room. The intervention was performed in 41% of the patients in the trabeculectomy group and 36% of the patients in the phacotrabeculectomy group. This is a relatively high rate and reflects the surgeon’s preference for needling of the bleb in theatres. It also reflects the intensive nature of the post-operative care required for both trabeculectomy and phacotrabeculectomy procedures to attain the desired IOP outcomes for these patients. Laser suture lysis and suture adjustments were also very common post-operative interventions required by patients in both surgical groups, with a similar rate of 23%.

While the rate of post-operative interventions was comparable between the two groups, there were more anterior chamber reformations in the phacotrabeculectomy group compared to the trabeculectomy group. This might represent a tendency on the part of the surgeon to allow more drainage through the scleral flap when performing phacotrabeculectomy in anticipation of a greater tendency for fibrosis in the post-operative period. Furthermore, the technique for titrating the flow during flap closure would have been slightly different between the two groups, as an anterior chamber maintainer was generally used for standalone trabeculectomy only.

Visual acuity was confounded by the effect of phacoemulsification. Perhaps not surprisingly the pre-operative VA was slightly worse in the phacotrabeculectomy group compared to the trabeculectomy group with a lower proportion having a BCVA of 6/6 or better and a higher proportion having a BCVA or worse than 6/18. For this reason, loss of VA was analyzed rather than any improvement in VA. A decrease in VA by two or more lines was similar between the trabeculectomy and phacotrabeculectomy groups at one year however differed after that. A greater proportion of patients had a decrease in VA of two or more lines in the trabeculectomy group at two years and five years. This may reflect more severe glaucoma (overall MD was higher in the trabeculectomy group, although not reaching statistical significance), or also could be linked to cataract progression in the phakic eyes, which is commonly associated with glaucoma surgery.

Of the phakic patients in the trabeculectomy group, 43% underwent phacoemulsification within five years of their initial procedure. The TVT study also found 43% of patients required phacoemulsification within five years after trabeculectomy [7]. It is noteworthy that patients who undergo phacoemulsification after trabeculectomy are also at higher risk of bleb failure [17].

In all glaucoma surgeries, the benefit of lowering IOP must be balanced against the risk of adverse events associated with the procedure. Intra-operative complications occurred at the same rate, with 9% of both trabeculectomy and phacotrabeculectomy groups having complications recorded. There was no significant difference in the rate of early post-operative complications between the two surgical groups. However, there was a higher rate of late post-operative complications in the trabeculectomy group compared to the phacotrabeculectomy group.

The early and late post-operative complication rates for the trabeculectomy group of this study are lower than both the TVT and PTVT studies, with an early post-operative complication rate of 27% and a late post-operative complication rate of 23%. However, methodological differences in the assessment of post-operative complications, such as the classification of which complications are included, might account for this.

The phacotrabeculectomy group had lower and less diffuse blebs, and required more post-operative medications, supporting the notion that the bleb morphology can be different in phacotrabeculectomy. The older age in the phacotrabeculectomy group may also explain the lower rates of encapsulation and needling because older patients have less Tenon’s tissue and are less likely to scar. One of the main issues with bleb-forming surgery is the potential for late post-operative infection which can be sight-threatening. There were no cases of post-operative infection during the five years of follow-up.

While the rate of treatment failure, post-operative interventions, and post-operative complications was similar in both groups, the trabeculectomy procedure had more late post-operative complications than the phacotrabeculectomy group. This most likely reflects the more prominent blebs present in these eyes, as well as the fact that this group had slightly more advanced glaucoma. Also, as the phacotrabeculectomy group is expected to heal more aggressively, perhaps post-operative complications, if they are to occur, will occur earlier in the post-operative period.

Few studies within the last ten years have compared trabeculectomy directly with phacotrabeculectomy with periods of follow up longer than two years. A recent study in 2019 examined two-year outcomes of trabeculectomy and phacotrabeculectomy and reported very similar findings to the current study [9]. The mean IOP after two years was 11.5 ± 3.1 in the trabeculectomy group and 12.5 ± 4.8 in the phacotrabeculectomy group [9]. The mean number of glaucoma medications was 2 ± 1 in both surgical groups at baseline and was reduced to 0.3 in the trabeculectomy group and 0.4 in the phacotrabeculectomy group after two years [9].

The treatment outcomes of trabeculectomy after five years of follow up in the TVT study show a mean IOP of 12.6 ± 5.9 mm Hg, 1.2 ± 1.5 glaucoma medications required [7]. Three years of results have been published for the PTVT which shows a mean IOP of 12.1 ± 4.8 mm Hg and 1.2 ± 1.5 glaucoma medications required [18]. When compared to this study’s trabeculectomy results at five years, there is a lower mean IOP of 10.6 ± 2.7 mm Hg and the number of glaucoma medications required of 0.3 ± 0.7.

Limitations of the study

This was a retrospective study design, and the two groups were not randomized. There was a statistically significant difference in age between the trabeculectomy and phacotrabeculectomy groups. The median age of the trabeculectomy group was 68 years as opposed to 78 years in the phacotrabeculectomy group, reflecting the fact that older glaucoma patients are more likely to have a concomitant cataract. The mean pre-operative IOP of the trabeculectomy group was 28.0 ± 9.8 as opposed to 23.4 ± 7.9 in the phacotrabeculectomy group which is also statistically significant. This could be linked to the younger median age in the trabeculectomy group as some of the younger patients in this cohort may have had more aggressive glaucoma. Furthermore, if patients had more aggressive glaucoma or higher pressures it may have been less likely for them to have been offered combined phacotrabeculectomy, especially if the cataract was relatively mild in slightly younger patients, and this represents a possible source of selection bias. The fact that patients in the phacotrabeculectomy group were on average older may also have biased the outcome as these patients would be expected to exhibit less post-operative scarring - although if they had been on glaucoma medications for longer than their younger counterparts (duration of medication use also being a significant risk factor for scarring) this could partially offset the effect of increasing age.

A major confounding factor that may have influenced results is subtle variations in technique between the two surgical groups, as well as the fact that case selection was at the surgeon’s discretion. It is always a dilemma choosing a surgical procedure for a patient with significant cataract and progressing glaucoma. Potentially those cases perceived to be at high risk for post-operative scarring may have been preferentially selected for trabeculectomy alone.

The surgical technique used for all cases followed the Moorfields Safer Surgery System [19]. Several steps in both trabeculectomy and phacotrabeculectomy procedures were standardized including the route of administration of anti-fibrotics and the general principles of the aforementioned trabeculectomy technique. There was slight variation between the trabeculectomy and phacotrabeculectomy procedures as for most of the standalone cases an anterior chamber (AC) maintainer was used as standard. In combined cases, an AC maintainer was rarely used as the pupil was often semi-dilated and there would be a significant risk of iris prolapse. This may have been a potential source of bias. Furthermore, the surgeon’s technique may have evolved over time, potentially having an impact on the outcomes of patients who had their operation at a later date.

This study is retrospective by design, despite the fact that data was collected in a contemporaneous fashion as follow-ups were occurring prior to the commencement of the study. Nevertheless, this introduces the possibility of selection bias, and there was also some selection bias with the non-standardized use of anti-fibrotics intra-operatively. Patients in the phacotrabeculectomy group were more likely to have received more MMC and in higher doses in anticipation of the perceived higher failure rate associated with the procedure [20]. It is also likely that slightly higher doses of MMC were used in the pseudophakic as compared to the phakic eyes undergoing standalone trabeculectomy, which may account for the somewhat counter-intuitive finding that success rates of standalone trabeculectomy in both phakic and pseudo-phakic eyes were similar.

There was a tendency towards lower IOP seen in the trabeculectomy group as compared to the phacotrabeculectomy group however this did not reach statistical significance. This was also shown by Sugimoto et al. in 2015 [21]. It is possible that a larger study may have reached statistical significance, and the present study may have been underpowered to detect this difference if, in fact, the difference is relatively small.

## Conclusions

This study describes five-year outcomes of eyes undergoing trabeculectomy and phacotrabeculectomy for medically uncontrolled glaucoma. There was a similar mean IOP and IOP reduction from baseline post-operatively between the trabeculectomy and phacotrabeculectomy groups. Safety, as measured by post-operative complication rates, and the need for post-operative interventions were similar. However, the rate of absolute success at five years was higher with trabeculectomy than with phacotrabeculectomy. In selected cases, phacotrabeculectomy can be a very useful option for managing patients with co-existing glaucoma and visually significant cataract. The IOP outcome was comparable, but with an increased chance of needing supplemental glaucoma medications and with some differences in bleb morphology.
